# An improved YOLOv8s-based UAV target detection algorithm

**DOI:** 10.1371/journal.pone.0327732

**Published:** 2025-08-21

**Authors:** Xinwei Wang, Yue Hu, Qing Liang, Yujie He, Li Zhou

**Affiliations:** 1 Xi’an University Of Posts And Telecommunications, School Of Electronic Engineering, Xi’an, Shaanxi Province, China; 2 Xi’an University Of Posts And Telecommunications, School of Communication and Information Engineering, Xi’an, Shaanxi Province, China; 3 Xi’an University Of Posts And Telecommunications, School of Automation, Xi’an, Shaanxi Province, China; Chandigarh University Institute of Engineering, INDIA

## Abstract

At present, the low-altitude economy is booming, and the application of drones has shown explosive growth, injecting new vitality into economic development. UAVs will face complex environmental perception and security risks when operating in low airspace. Accurate target detection technology has become a key support to ensure the orderly operation of UAVs. This paper studies UAV target detection algorithm based on deep learning, in order to improve detection accuracy and speed, and meet the needs of UAV autonomous perception under the background of low altitude economy. This study focuses on the limitations of the YOLOv8s target detection algorithm, including its low efficiency in multi-scale feature processing and insufficient small target detection capability, which hinder its ability to perform rapid and accurate large-scale searches for drones. An improved target detection algorithm is proposed to address these issues. The algorithm introduces AKConv into the C2F module. AKConv allows for convolution kernels with arbitrary numbers and sampling shapes, enabling convolution operations to more precisely adapt to targets at different locations, thereby achieving more efficient feature extraction. To further enhance the model’s ability to extract critical features of small targets, the SPPF module incorporates the LSKA mechanism. This mechanism captures long-range dependencies and adaptivity more effectively while addressing computational complexity issues associated with large convolution kernels. Finally, the Bi-FPN feature pyramid network structure is introduced at the 18th layer of the model to accelerate and enrich feature fusion in the neck. Combined with the SCDown structure, a novel Bi-SCDown-FPN feature pyramid network structure is proposed, making it more suitable for detecting targets with insufficient feature capture in complex environments. Experimental results on the VisDrone2019 UAV dataset show that the improved algorithm achieves a 5.9%, 4.5%, and 6.1% increase in detection precision, detection recall, and mean average precision, respectively, compared to the original algorithm. Moreover, the parameter count and weight file size are reduced by 13.41% and 13.33%, respectively. Compared to other mainstream target detection algorithms, the proposed method demonstrates certain advantages. In summary, the target detection algorithm proposed in this paper achieves a dual improvement in model lightweighting and detection accuracy.

## 1. Introduction

With the continuous innovation and progress of aviation technology, UAVs have been widely used in many fields [1]. The UAV target detection task utilizes computer vision and other technologies to enable the UAV to identify ground targets in the low-altitude environment, so as to perceive the surrounding environment more effectively [2]. This technology supports UAVs to play an important role in search and rescue, surveillance and patrol, aerial photography and other tasks [3]. However, UAV-based target detection faces many challenges, mainly due to its complex operating environment and changing application scenarios [4].

First, drones often perform tasks in highly dynamic environments, and their rapid changes in speed and angle place high demands on detection algorithms [2]. During flight, the perspective and size of the target constantly change, especially in complex terrains or during high-speed movements, where targets may frequently scale or move within the frame, causing traditional object detection algorithms to fail or reduce in accuracy [5]. Secondly, complex background interference is also a major challenge in drone-based object detection [6]. The scenes in which drones operate are diverse, with backgrounds containing elements like trees, buildings, and vehicles, which can easily cause confusion between targets and the background [7]. Additionally, obstacles in the natural environment may partially obscure targets, making it difficult for detection algorithms to identify them, thus increasing the risks of false positives and missed detections [8]. Therefore, improving the detection accuracy and lightweight nature of models for drones in complex scenarios is crucial [9].

Currently, deep learning-based object detection is mainly divided into two categories: region-based object detection and regression-based object detection [10]. Region-based object detection methods, such as Region-based Convolutional Neural Network(R-CNN) and its variants Fast Region-based Convolutional Neural Network (Fast R-CNN) [11] and Faster Region-based Convolutional Neural Network (Faster R-CNN) [12], generate candidate regions and then perform feature extraction and classification for each region. The advantage of this method is its ability to achieve high detection accuracy, especially in complex backgrounds [13]. On the other hand, regression-based object detection methods, such as the You Only Look Once (YOLO) [14] series and Single Shot MultiBox Detector (SSD) [15], divide the entire image into grids and directly predict the location and category of objects in each grid. Compared to region-based methods, these algorithms have higher detection speed and can achieve real-time processing, making them suitable for scenarios requiring rapid response [16].

There have been some research achievements in object detection, with improvements that enhance the drone’s detection capabilities in complex environments, thus supporting broader practical applications [17]. Literature [18] proposes a lightweight anti-UAV detection model in order to solve a series of security risks of UAVs in civil and military scenarios. By introducing Ghost convolution at the neck of the model to reduce the model size, The structure of the model is optimized by adding the Efficient Multi-scale Attention (EMA) module and using the Deformable Convolutional Netv2(DCNv2) to improve the detection headers. In literature [19], airborne edge devices on UAVs are used to detect objects from different perspectives, and a Lightweight object detection algorithm based on YOLOv8n is proposed. Through the design of Lightweight HGNet (LHGNet) backbone network, the depth can be separated convolution and channel mixing module is integrated to dig deep inner features. In addition, the Lightweight GS(LGS) bottleneck layer and Lightweight GS Cross-Stage Partial (LGSCSP) fusion module are introduced in the neck to reduce the computational complexity. Finally, the ability of the model to capture small targets is enhanced by modifying the structure and the size of the feature map. Literature [20] proposed a monitoring system for early detection of forest fires. The detection and classification performance of YOLOv8 and YOLOv5 were compared by means of images collected by a camera mounted on a quad-rotor UAV. The CNN-RCNN classification network was also constructed, and real-time detection was realized based on NVIDIA Jetson Nano. The test results show that the accuracy of various methods is 89% to 96%. Literature [2] mainly studies the small target detection technology of UAV aerial images, and proposes a multi-scale detection method based on Adaptive Feature fusion. By adding Adaptive Feature Extraction Module (AFEM) to the backbone network, the adaptive Feature Extraction module can adjust the convolutional kernel receptor field and reduce redundant background. Adaptive Feature Weighted Fusion Network (SBiFPN) is designed to enhance the shallow feature expression of small targets, increase the detection scale of small targets, and expand the receptive field. Literature [21] In order to cope with the challenges such as insufficient accuracy and detection speed of UAV aerial image target detection algorithm, A Lightweight Feature Extraction Reparameterised Efficient Layer Aggregation Network (LFERELAN) module is proposed. It is designed to enhance the extraction of small target features and optimize the use of computing resources; The Lightweight Cross-scale Feature Pyramid Network (LC-FPN) is also used to further enrich the feature information. Finally, the Shared Convolution Detection Head Lightweight, Detail-enhanced and shared convolution detection head (LDSCD-Head) are proposed to optimize the original detection head. Literature [22] proposed a detection model based on YOLOv8 to solve the problem of missing and false detection in UAV aerial infrared image target detection, which improved the backbone feature extraction network based on Ghost and HGNetv2(GhostHGNetV2). Coordinate Attention (CoordAtt) is introduced in the neck, and the channel dimension of the feature map is weighted to improve the detection accuracy and robustness. XIoU is used as the bounding frame loss function to enhance the target positioning accuracy.

To sum up, although some progress has been made in the field of UAV target detection, there are still challenges to achieve high-precision and high-speed detection in complex scenarios, and it is difficult for existing methods to effectively improve the adaptability and real-time detection ability of models in complex environments while taking into account detection accuracy. At present, there are relatively few systematic studies on these key issues, which is exactly the research gap that this study aims to fill.

In order to further improve the accuracy of target detection, optimize the network structure and realize the lightweight of the network [23], this paper selects YOLOv8s as the basic network for research. Because our research focused on low-altitude UAV target detection, the target size in the dataset is small and the shape is complex and diverse. Compared with the latest models such as YOLOv9 and YOLOv10, YOLOv8s has a relatively lightweight structure, which can effectively capture the features of small targets when extracting multi-scale features. In addition, this study needs to realize real-time detection under the limited computing resources of UAVS, and the parameter number and computation amount of YOLOv8s are relatively low, while the newer versions of YOLOv9 and YOLOv10 models are more complex. Therefore, the main work of this paper based on YOLOv8s is as follows:

Alterable Kernel Convolution (AKConv) is introduced into the C2F module, replacing the original conventional convolution module with AKConv to construct a new C2F_AKConv module. This replaces some of the C2F modules in the backbone and neck networks, achieving lightweighting while continuing to improve accuracy. The C2F_AKConv module consists of the C2F and AKConv modules, which significantly enhance the model’s ability to capture contextual information in the YOLOv8s network.

In drone-based object detection tasks, due to the complex and diverse ground backgrounds and changing environmental conditions, there is a need to further improve the model’s ability to extract key features of small targets [24]. This paper introduces the Large Separable Kernel Attention (LSKA) attention mechanism into the Spatial Pyramid Pooling Fast(SPPF) [25] module of YOLOv8s to optimize the model’s performance, better capture long-range dependencies and adaptiveness, and effectively address the computational complexity when handling large convolution kernels.

Finally, a Bidirectional Feature Pyramid Network (Bi-FPN) structure is introduced at the 18th layer of the model to accelerate and enrich the fusion of neck features. Combined with the Spatial Convolution Downsample (SCDown) structure, a new Bi-SCDown-FPN feature pyramid network structure is proposed, applying the concept of Bi-FPN feature fusion to YOLOv8s and replacing conventional convolution modules in the neck network with the SCDown module. This redesigns the neck feature pyramid network structure of YOLOv8s to make it more suitable for object detection in complex environments where feature capture is insufficient.

## 2. YOLOv8s algorithm

The YOLOv8 series is a further development of previous versions such as YOLOv5 [26] and YOLOv6 [27], aimed at enhancing the model’s performance and adaptability. The YOLOv8 series offers multiple versions of different sizes, including YOLOv8n, YOLOv8s, YOLOv8m, YOLOv8l, and YOLOv8x, each with its own characteristics in terms of algorithm details, performance, and application scenarios. Among these, YOLOv8s is the small version, specifically designed to provide excellent performance under lightweight requirements. Its main goal is to address the needs of resource-constrained devices, making it suitable for scenarios with limited computing power and storage space, such as embedded systems, edge computing devices, and drones [28].

YOLOv8s achieves a balance between high object detection accuracy and significantly reduced computational complexity and power consumption through optimization of its network structure and parameter simplification. This allows it to perform exceptionally well in tasks with high real-time requirements [29]. For scenarios that require rapid response and efficient detection, YOLOv8s is a good choice, balancing detection speed and accuracy under limited resources [30]. The YOLOv8s network structure is mainly composed of four parts: the input layer, backbone network, neck network, and prediction network.

The input side is responsible for receiving the input image and performing key preprocessing operations. Image sizing is usually done by using bilinear interpolation or more advanced algorithms to scale the image uniformly to a specific size suitable for network processing to ensure that the model can carry out consistent feature extraction operations for images from different sources. The normalization process is to map the image pixel value to a specific range such as [0, 1] or [−1, 1], which not only speeds up the convergence process of the model, but also enhances the generalization adaptability of the model to all kinds of images. Through this pre-processing process, the model can efficiently analyze the image information, lay a solid foundation for the subsequent feature extraction and target detection, and significantly accelerate the training and reasoning speed, effectively guarantee the accuracy and reliability of the final detection results.

The backbone network is mainly responsible for feature extraction, adopts the Darknet-53 framework, and introduces a new C2F module to carry out residual learning. Conv module parameters such as convolution kernel size, step size and filling are carefully designed to balance the feature extraction effect and computational effort. The module draws on the design of Cross Stage Partial(CSP) [31] and (Efficient Layer Aggregation Network)ELAN, by adding a large number of skip connections and additional Split operations, The gradient information generated by the front-end network is efficiently and accurately integrated into the back-end feature map, alleviating the problem of gradient disappearance and enabling the network to learn more in-depth features. During the specific construction, Conv convolutional module and C2F module are sequentially superimposed four times, and each completed superposition is defined as a stage. Finally, the model adopts the SPPF module similar to the YOLOv5 architecture, and through three consecutive 5 × 5 maximum pooling operations, the vector sizes of feature graphs at different scales are normalized to a uniform specification, providing consistent and stable feature input for subsequent processing.

The neck network is responsible for feature fusion. It replaces the CSP Bottleneck with three convolutions(C3) module with the C2F module and combines the concepts of Path Aggregation Network (PAN) and Feature Pyramid Network (FPN) to build an up-and-down feature pyramid structure. Additionally, YOLOv8 removes the 1 × 1 convolution operation before upsampling that was present in YOLOv5 and YOLOv6, directly upsampling the feature maps from each stage of the backbone. This simplified operation not only improves the simplicity of the network structure, but also significantly improves the efficiency and quality of feature fusion.

The prediction network transitions from the anchor-based coupling head in YOLOv5 to an anchor-free decoupled head. This structure no longer includes the objectness branch, but instead, it has decoupled classification and regression branches, with the latter using Distributive Focal Loss (DFL). This design makes the model more flexible and accurate in predicting the target category and location, effectively reduces the coupling interference between tasks, and greatly improves the detection efficiency and accuracy.

**Fig 1:**
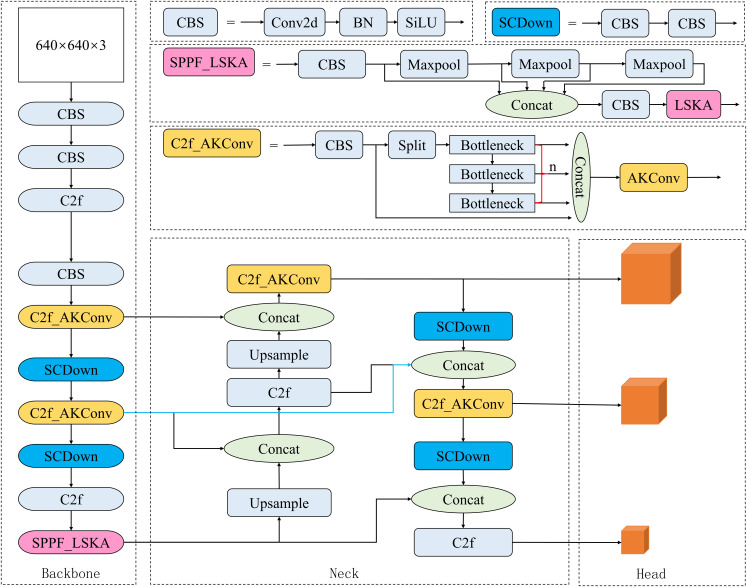
Structure diagram of the optimized YOLOv8s model. The three improved parts are C2F_AKConv in orange, SPPF_LSKA in pink and SCDown in blue, and the blue line segment is the Bi-FPN feature pyramid network structure.

To address the issues with detection speed and accuracy in the YOLOv8s object detection algorithm, this paper proposes several improvements to the network model. First, AKConv is introduced into the C2F module in the backbone network. Then, the LSKA attention mechanism is incorporated into the SPPF module in the backbone network. Finally, the Bi-FPN feature pyramid network structure is introduced at the 18th layer of the model, as indicated by the blue line segments in Fig 1, and combined with the SCDown structure. The optimized YOLOv8s model structure is shown in Fig 1.

## 3. Experimental theory

### 3.1 Incorporating alterable Kernel convolution AKConv

In the field of deep learning, traditional convolution operations have two main shortcomings. First, convolution operations run on fixed-size windows, which limits their ability to capture information from other windows. Additionally, the shape of the window is fixed, restricting the model’s capacity to extract features at different scales and shapes. Second, the size of the convolution kernel is fixed at k × k, meaning that the window size is also fixed. As k increases, the number of parameters grows rapidly, leading to a significant increase in computational complexity.

To address the two limitations of traditional convolution operations, AKConv allows for the use of arbitrary shapes and an arbitrary number of parameters, enabling the convolution kernel to adapt flexibly to the different needs of the input features. Through an initial sampling coordinate algorithm and adaptive sampling position adjustments, AKConv can adapt to different targets while supporting linear scaling of convolution parameters to optimize lightweight models. In practical applications, when performance needs to be improved, a larger convolution kernel can be chosen, while a smaller kernel can be selected to achieve a more lightweight model.

Convolutional neural networks rely on convolution operations, which locate features at corresponding positions through a regular sampling grid. The regular sampling grid for a 3 × 3 convolution operation is shown in equation (1), where *R* represents the sampling grid.


R={(−1,−1),(−1,0),…(0,1),(1,1)} (1)

However, AKConv requires the use of an irregular sampling grid, so an algorithm is designed to generate the initial sampling coordinates for the convolution kernel *P*_*n*_. Since irregular convolutions rarely have a central point in terms of size, to adapt to the convolution size being used, the sampling origin is set at (0,0) at the top-left corner of the algorithm. The corresponding convolution operation at position *P*_*0*_ is defined as follows, where the convolution parameter is denoted as ω.


$$Conv\left(P_{0}\right)=\sum \omega \times \left(P_{0}+P_{n}\right)$$ (2)

**Fig 2:**
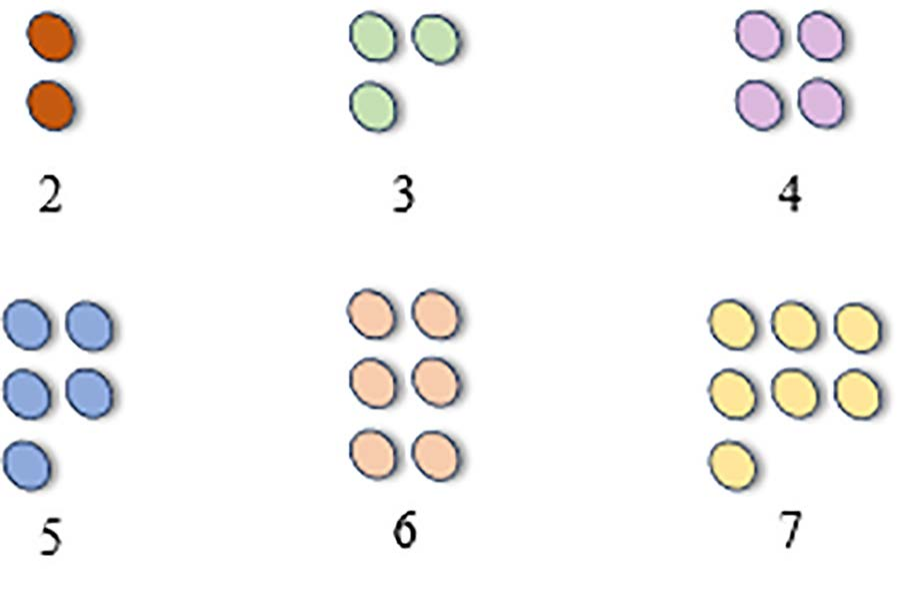
Convolution kernel of any size.

**Fig 3:**
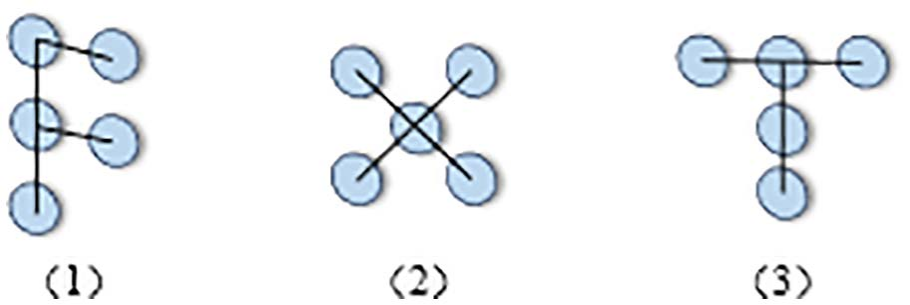
Convolution kernel of size 5 with arbitrary shape.

Through a series of operations, AKConv effectively addresses the issue caused by irregular sampling coordinates that cannot match the corresponding size of convolution operations. Fig 2 illustrates the generation of initial sampling coordinates for convolutions of any size, while Fig 3 demonstrates a convolution kernel of size 5 with an arbitrary shape.

**Fig 4:**
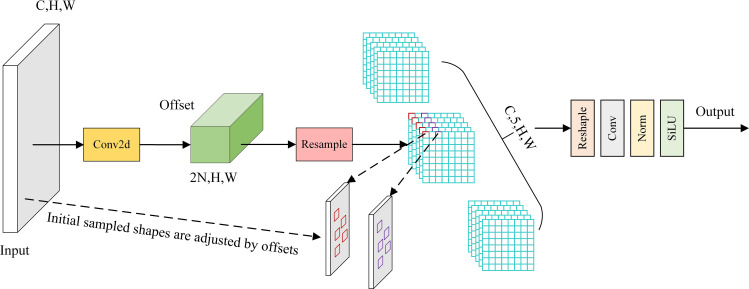
AKConv module structure.

For the input sampling coordinates (C, H, W), the convolution kernel’s offset is first obtained through a 2D convolution operation. Then, the offset is added to the original coordinates to obtain the corrected coordinates (2N, H, W), where N is the size of the convolution kernel. Next, the corresponding features at the adjusted positions are obtained through linear interpolation and resampling. Afterward, the features are further adjusted, convolved, normalized, and finally passed through the Sigmoid Linear Unit (SiLU) activation function to generate the feature map. The structure of the AKConv module is shown in Fig 4.

### 3.2 Introduction of LSKA attention mechanism

**Fig 5:**
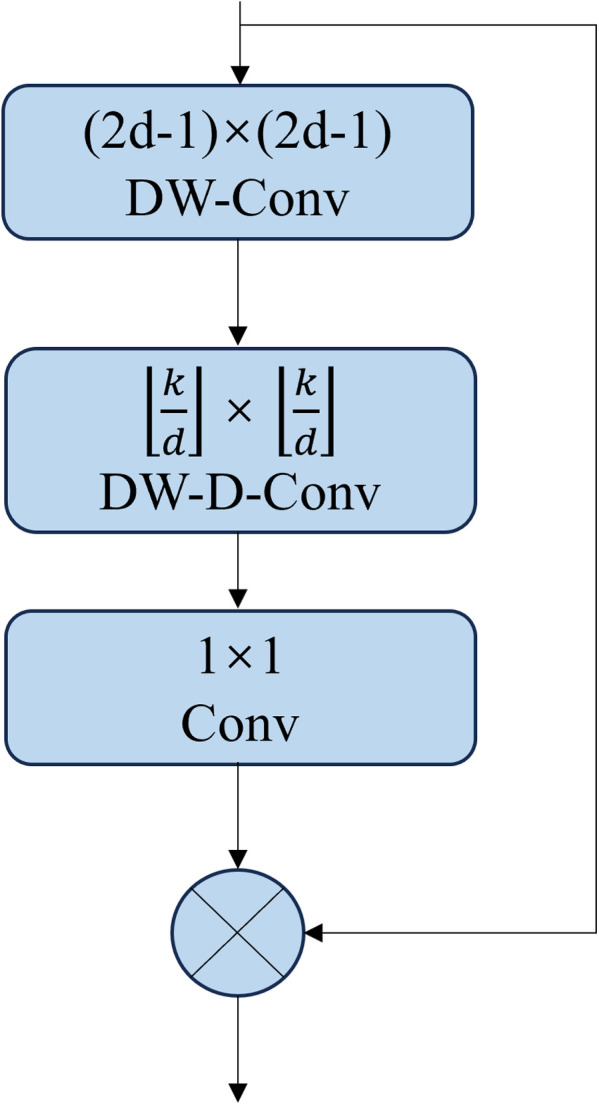
LKA Network Structure.

The LKA (Large Kernel Attention) mechanism decomposes a large convolution kernel into depthwise convolution, dilated convolution, and pointwise convolution. This decomposition method retains the wide receptive field characteristic brought by the large convolution kernel, while effectively reducing the number of parameters in the model, thereby achieving model lightweighting. The structure of LKA is shown in Fig 5.

Given the feature *F ∈ R*^*H×W×C*^, where *H* is the height of the channel, *W* is the width of the channel, and *C* is the number of input channels, the implementation of LKA is shown in equation (3).


$$\left\{ \begin{array}{c} {\bar{Z}}^{C}=\sum {\mathrm{\backslash nolimits}}_{H,W}W_{(2d-1)\times (2d-1)}^{C}*F^{C}\\ Z^{C}=\sum {\mathrm{\backslash nolimits}}_{H,W}W_{\left\lfloor \frac{k}{d}\right\rfloor \times \left\lfloor \frac{k}{d}\right\rfloor }^{C}*{\bar{Z}}^{C}\\ A^{C}=W_{1\times 1}*Z^{C}\\ {\bar{F}}^{C}=A^{C}⊗F^{C} \end{array}\right.$$ (3)

In this context, * represents convolution, ⊗ represents the Hadamard product, and ${\bar{Z}}^{C}$ denotes the convolution output with a kernel size of (2d−1)×(2d−1),used to compensate for the grid effect caused by $Z^{C}$. $Z^{C}$ represents the convolution output with a kernel size of $\left\lfloor \frac{k}{d}\right\rfloor \times \left\lfloor \frac{k}{d}\right\rfloor$, *k* represents the maximum receptive field of the convolution kernel, where *d* is the dilation rate. ⌊ ⋅ ⌋ denotes the floor operation. Next, a 1 × 1 convolution is applied to obtain the attention map $A^{C}$; finally, the attention map$A^{C}$ is used in the Hadamard product with the input feature map $F^{C}$ to obtain the output feature map ${\bar{F}}^{C}$.

**Fig 6:**
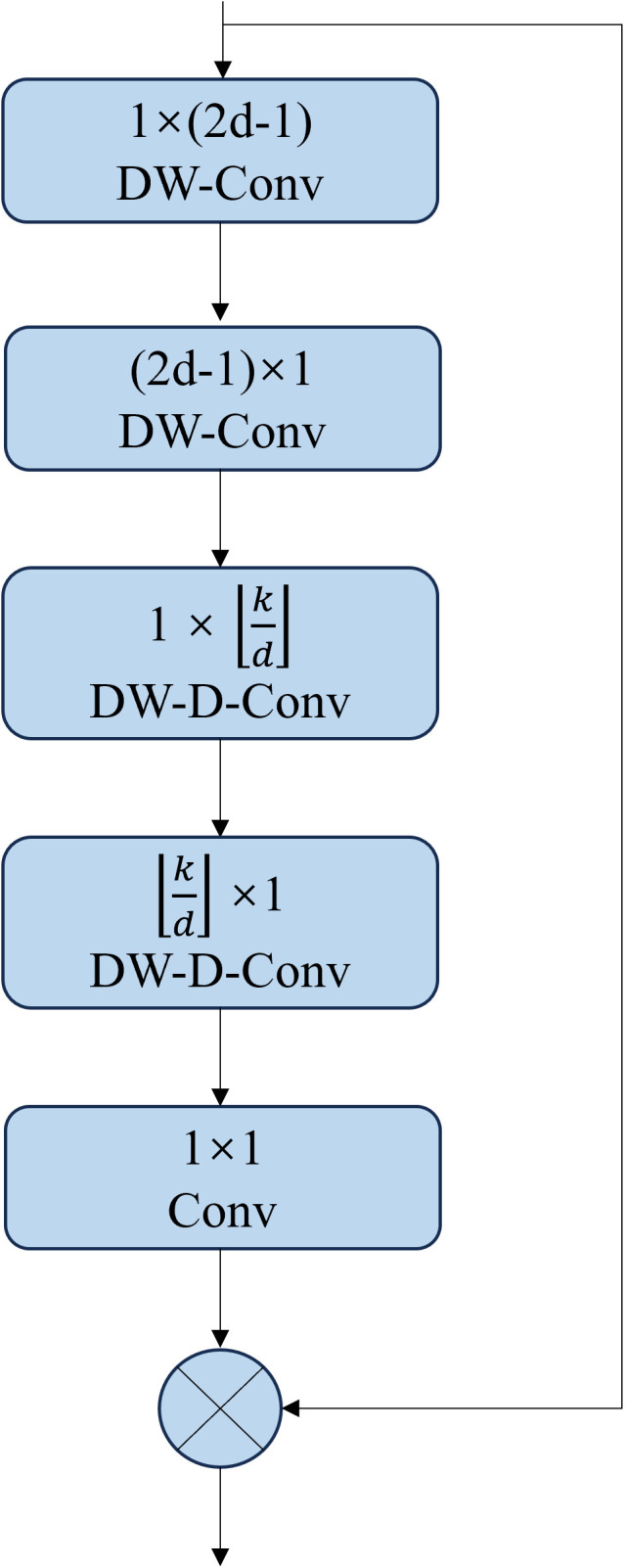
LSKA Network Structure.

However, in LKA, the convolution kernel grows quadratically. To reduce computational complexity, a large separable kernel attention module LSKA is introduced. LSKA further decomposes the original LKA’s 2D depthwise convolution kernel and depthwise dilated convolution kernel into 1D horizontal and vertical convolution kernels, and then these decomposed kernels are concatenated. This process effectively reduces computational overhead and memory requirements while improving the model’s ability to capture long-range dependencies and adaptability. The structure of LSKA is shown in Fig 6.

At the same time, the implementation of LSKA is also changed from equation (3) to equation (4).


$$\left\{ \begin{array}{c} {\bar{Z}}^{C}=\sum {\mathrm{\backslash nolimits}}_{H,W}W_{(2d-1)\times 1}^{C}*\left(\sum {\mathrm{\backslash nolimits}}_{H,W}W_{1\times (2d-1)}^{C}*F^{C}\right)\\ Z^{C}=\sum {\mathrm{\backslash nolimits}}_{H,W}W_{\left\lfloor \frac{k}{d}\right\rfloor \times 1}^{C}*\left(\sum {\mathrm{\backslash nolimits}}_{H,W}W_{1\times \left\lfloor \frac{k}{d}\right\rfloor }^{C}*{\bar{Z}}^{C}\right)\\ A^{C}=W_{1\times 1}*Z^{C}\\ {\bar{F}}^{C}=A^{C}⊗F^{C} \end{array}\right.$$ (4)

Finally, a comparative analysis of the performance metrics measuring computational complexity in LKA and LSKA is conducted, including the number of parameters (Params) and floating-point operations (FLOPs). Assuming the input and output feature maps are of sizeH×W×C, the comparison results are shown in Table 1.

**Table 1:** Comparison of Computational Complexity Between the Two Models.

Models	Params	FLOPs
LKA	${\left(2d-1\right)}^{2}\times C+{\left\lfloor \frac{k}{d}\right\rfloor }^{2}\times C+C\times C$	$\left({\left(2d-1\right)}^{2}\times C+{\left\lfloor \frac{k}{d}\right\rfloor }^{2}\times C+C\times C\right)\times H\times W$
LSKA	$\left(2d-1\right)\times C\times 2+\left\lfloor \frac{k}{d}\right\rfloor \times C\times 2+C\times C$	$\left(\left(2d-1\right)\times C\times 2+\left\lfloor \frac{k}{d}\right\rfloor \times C\times 2+C\times C\right)\times H\times W$

By comparing the computational expressions, it can be seen that when *d* > 1, the coefficients of the parameter count and the floating-point operations for LSKA are smaller, making LSKA’s computational complexity lower than that of LKA. Therefore, LSKA is more suitable for lightweight detection models, as it significantly reduces computational resource consumption while maintaining performance.

### 3.3 Proposed Bi-SCDown-FPN feature pyramid network structure

**Fig 7:**
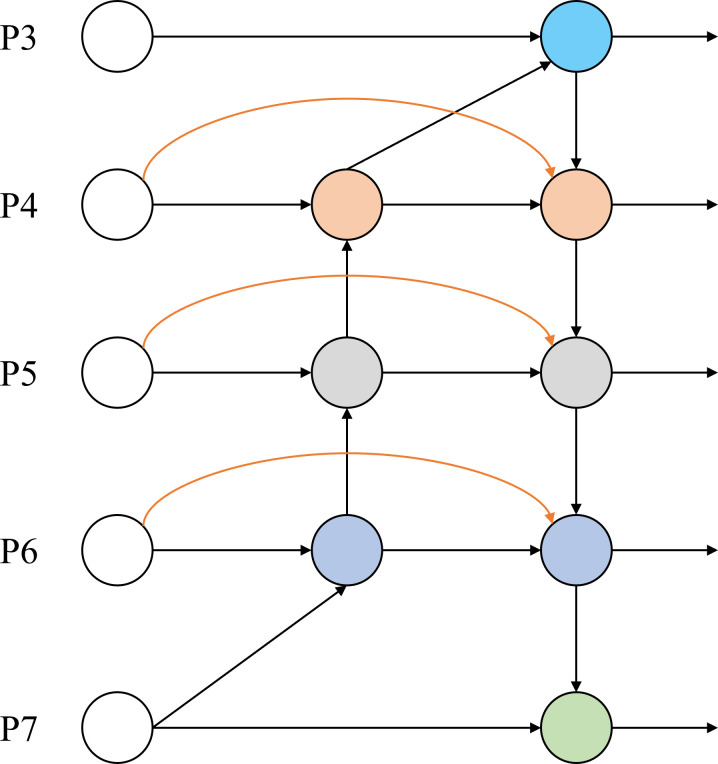
BiFPN Structure Diagram. There are 5 levels of nodes from P3 to P7. The straight line represents the feature transmission path, and the orange arc represents the bidirectional connection relationship, showing the flow and fusion of features between different levels.

Efficient multi-scale feature fusion methods, such as FPN, PANet, and Neural Architecture Search – Feature Pyramid Network (NAS-FPN), assume that the contributions of each feature layer to the output are the same when integrating different input features. However, due to differences in the resolution of these input features, their importance to the output may not be consistent. To address this issue, a simple yet efficient weighted bidirectional feature pyramid network BiFPN is proposed. The structure of BiFPN is shown in Fig 7.

BiFPN makes two main improvements in feature fusion. First, in PANet, feature fusion is achieved through concatenation (Concat) operations, which results in equal contributions from high-level and low-level features, without distinguishing the importance of features at different resolutions. In contrast, BiFPN introduces a set of learnable normalization weight parameters, allowing the model to automatically adjust the contribution of features from different layers based on data during training. This enables more flexible and effective feature fusion.Second, BiFPN achieves higher-dimensional feature fusion by stacking multiple layers. Compared to single-layer fusion, stacking multiple BiFPN modules can capture richer feature information, enhancing the model’s expressive power and improving performance in tasks such as object detection. Based on this, three weighted fusion methods are proposed:

(1) **General Fusion:**

$$O=\sum {\mathrm{\backslash nolimits}}_{i}W_{i}\cdot I_{i}$$ (5)

Where, $W_{i}$ is the weight parameter of the convolution kernel, $I_{i}$ represents the pixel value or feature value of the input image or feature map, and O represents the output value.

(2) **Softmax-based Fusion:**

$$O=\sum_{i}\frac{e^{w_{i}}}{\sum_{j}e^{w_{j}}}\cdot I_{i}$$ (6)

Softmax is applied to each weight, normalizing all weights into a probability between 0 and 1, indicating the importance of each input. However, the additional Softmax operation can slow down the GPU, which leads to the introduction of a fast normalization fusion method.

(3) **Fast Normalization Fusion:**

$$O=\sum_{i}\frac{w_{i}}{\varepsilon +\sum_{j}w_{j}}\cdot I_{i}$$ (7)

By applying the ReLU function after each $w_{i}$ to ensure $w_{i}\geq 0$,ε = 0.0001 to avoid numerical instability. Similarly, all weights are normalized between 0 and 1, but without the Softmax operation, making this method much more efficient. BiFPN uses the fast normalization fusion method.

**Fig 8:**
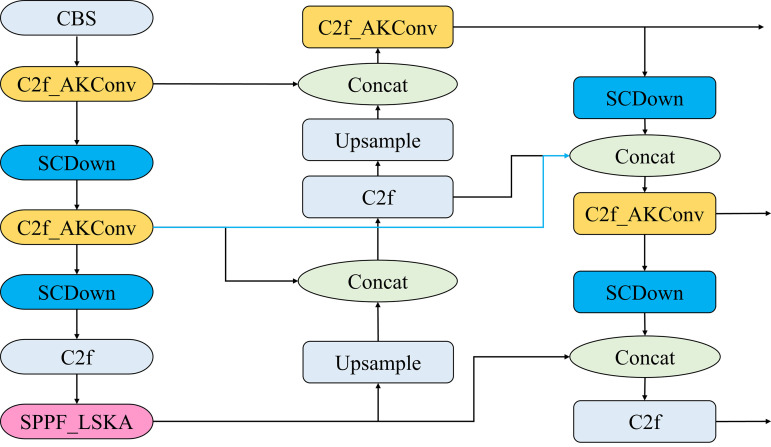
Bi-SCDown-FPN Structure Diagram.

**Fig 9:**
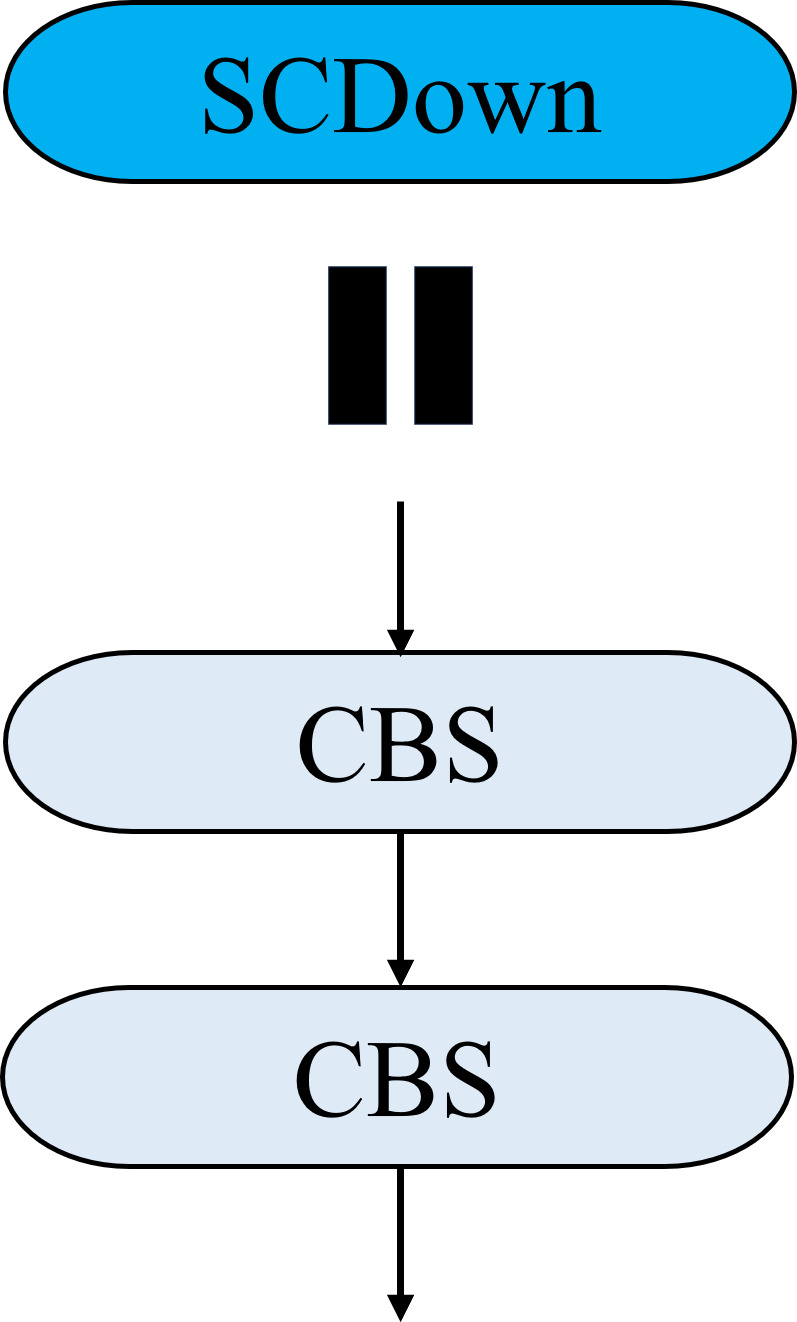
SCDown Module Structure Diagram.

The Bi-FPN feature pyramid network structure is introduced at layer 18 of the model, as shown by the blue lines in Fig 8. Additionally, the SCDown module replaces the conventional convolution module in the neck network, leading to a brand-new Bi-SCDown-FPN feature pyramid network structure. SCDown decouples spatial and channel dimensions, first adjusting the number of channels through a 1 × 1 pointwise convolution, and then applying a 3 × 3 depthwise convolution for spatial downsampling, minimizing computational cost while retaining maximum information. The SCDown module is shown in Fig 9.

## 4. Experiment results and discussion

### 4.1 Experiment environment setup

The hardware, software, and environment conFIGuration information for this experiment is shown in Table 2.

**Table 2:** Environment ConFIGuration Information.

Hardware ConFIGuration	Model
Operating System	Windows 11
CPU	R7-7840H
Memory	16GB
GPU	NVIDIA RTX 4060
Development Environment	Python 3.9, Pytorch 1.10, CUDA 11.3

The model training parameters are set as shown in Table 3.

**Table 3:** Parameter Settings.

Parameter Name	Parameter Size
Batch Size	16
Training Epochs	300
Weight Decay Coefficient	0.0005
Initial Learning Rate	0.001
Input Image Size	640 × 640

### 4.2 Dataset and evaluation metrics

**Fig 10:**
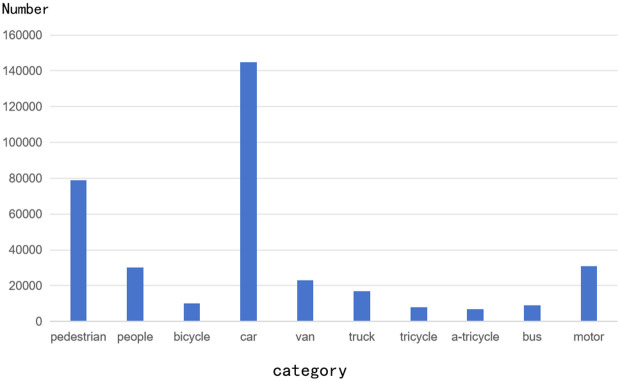
Target Sample Statistics of VisDrone2019.

The dataset used in this study is the VisDrone2019 dataset, released by the Machine Learning and Data Mining Laboratory at Tianjin University. The VisDrone2019 dataset is selected to enhance model generalization due to its large scale and diverse scenes, targets and illumination. Good labeling quality can reduce error interference; The data format is adapted to common tools, and the processing is convenient; Suitable for specific UAV missions, can optimize performance; And the community is rich in resources, which is conducive to rapid research. This dataset contains 10,209 high-altitude images taken by drones, covering various climatic conditions, shooting angles, and lighting variations. The dataset is divided into a training set with 6,471 images, a validation set with 548 images, and a test set with 3,190 images. It includes 10 categories, such as pedestrians, people, bicycles, cars, vans, trucks, tricycles, covered tricycles, buses, motorcycles. To evaluate the performance of the improved model, the VisDrone2019 object detection dataset was used for testing. Additionally, the performance of the improved model was compared with that of the original model on this dataset. As shown in Fig 10, the target sample statistics in the VisDrone2019 dataset are illustrated.

**Fig 11:**
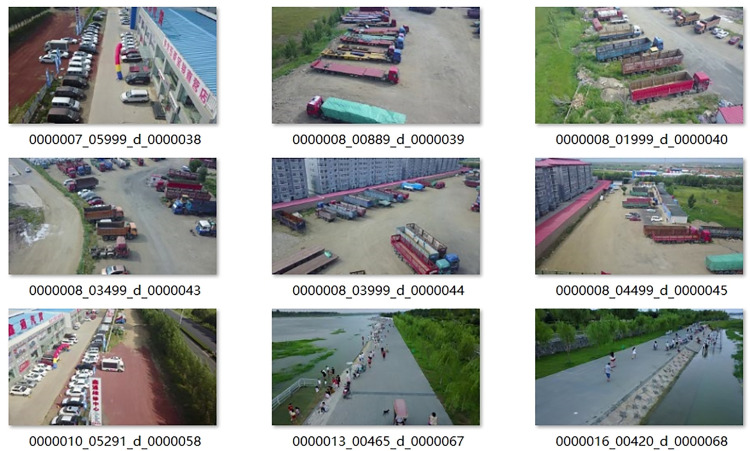
Partial Training Images of the Dataset.

As shown in Fig 11, these are some training images from the VisDrone2019 dataset.

To accurately evaluate the effectiveness of algorithm improvements, we analyze from two aspects: detection performance and lightweight metrics. We select Parameters (Params) and weight file size as indicators for lightweight evaluation, while Precision, Recall, and mean Average Precision (mAP_0.5) are used to assess detection accuracy.

Params represent the number of model parameters, reflecting the consumption of computational memory resources. The weight file size refers to the storage size of the trained model.

Precision (*P*) indicates the proportion of correctly identified objects among all detected objects, i.e., how many of the detected objects are true targets. Recall (*R*) represents the proportion of correctly identified targets among all actual targets, i.e., how many of the true targets have been successfully detected. mAP_0.5 denotes the mean Average Precision across all target classes when the IoU threshold is 0.5. The calculations for Precision and Recall are shown in Equations (8) and (9), respectively. The speed can be measured in terms of FPS, FPS represents the number of image frames a model can process per second.


$$P=\frac{TP}{TP+FP}$$ (8)

$$R=\frac{TP}{TP+FN}$$ (9)

In this context, True Positives (*TP*) refers to instances where the model successfully detects the target. False Positives (*FP*) refers to instances where the model incorrectly predicts a non-target as a target. False Negatives (*FN*) refers to cases where the model fails to detect the target. The Average Precision (*AP*) value reflects the trade-off between detection accuracy and recall at different confidence thresholds. The mean Average Precision (*mAP*) is calculated by averaging the *AP* values across all classes, as shown in Equations (10) and (11).


$$AP=\int_{0}^{1}p\left(r\right)d\left(r\right)$$ (10)

$$mAP=\frac{1}{N}\sum_{i=1}^{N}AP_{i}$$ (11)

Among them, *AP* in formula (10) is an integral calculation of accuracy in the range of recall rate from 0 to 1, which is used to measure the detection performance of the model for a single category. *N* is the total number of classes. Add all classes together and divide by the total number of classes *N*. The result is *mAP* of average accuracy means.

### 4.3 Training results

**Fig 12:**
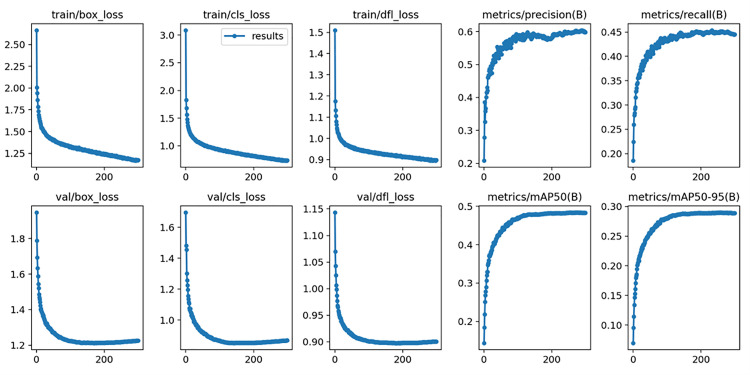
Improved training results of YOLOv8s.

As can be seen from Fig 12, the performance of the improved YOLOv8s model gradually converges and becomes stable after 300 training rounds on the VisDrone2019 data set. This result further confirms the effectiveness of the improved model.

### 4.4 Ablation experiment

In order to verify the effectiveness of the improved algorithm in this paper, ablation experiments were conducted on the VisDrone2019 data set by combining multiple modules. The evaluation indexes included five aspects: detection accuracy, detection recall rate, average detection accuracy, parameter number and weight file size, where √ indicates the introduction of the corresponding improvement strategy module. × indicates that no corresponding module is used. The ablation experiment results are shown in Table 4.

**Table 4:** Results of ablation experiment.

model	AKConv	LSKA	BiFPN	P (%)	R (%)	mAP (%)	Para (M)	Size (MB)	Speed (FPS)
model1	×	×	×	53.9	40.6	42.4	1.11	22.5	103
model2	√	×	×	56.8	42.6	45.1	1.05	21.2	167
model3	√	√	×	57.7	43.9	46.2	1.15	23.3	164
model4	√	√	√	59.8	45.1	48.5	0.96	19.5	156

According to the results of the ablation experiment in Table 4, model 1 serves as the benchmark model, adopting the original YOLOv8s algorithm without introducing any improved modules. Model 2 significantly enhances the detection performance by introducing the AKConv module into some C2F modules of the backbone network and the neck network. Compared with model 1, its detection accuracy, recall rate, average detection accuracy, and detection speed have increased by 2.9%, 2.0%, 2.7%, and 64 fps respectively. At the same time, the number of parameters and the size of the weight file have been reduced, decreasing by 6.05% and 5.78% respectively, demonstrating the remarkable effect of AKConv in improving the feature extraction ability and lightweighting the model.

Based on model 2, model 3 introduces the LSKA attention mechanism into the SPPF module of the backbone network, further increasing the detection accuracy, recall rate, and average detection accuracy by 0.9%, 1.3%, and 1.1% respectively. Although the number of parameters, the size of the weight file, and the detection speed have not been further reduced, the robustness of the model in complex scenarios has been enhanced.

Model 4 further combines the BiFPN structure and the SCDown module. Compared with model 3, the detection speed has decreased by 8 fps, but the detection accuracy, recall rate, and average detection accuracy have increased by 2.1%, 1.2%, and 2.3% respectively. It has also achieved significant lightweighting, with the number of parameters and the size of the weight file reduced by 16.39% and 16.31% respectively. Overall, compared with the benchmark model 1, model 4 has increased the detection accuracy, recall rate, average detection accuracy, and detection speed by 5.9%, 4.5%, 6.1%, and 53 fps respectively, while the number of parameters and the size of the weight file have decreased by 13.41% and 13.33% respectively.

In conclusion, the ablation experiment clearly shows that the AKConv module has a significant effect on the feature extraction ability and the lightweighting of the model. The LSKA attention mechanism effectively enhances the robustness of the detection accuracy. The combination of the BiFPN and SCDown modules, while maintaining the performance improvement, has achieved a further reduction in the number of parameters and the amount of calculation. These improvement points jointly verify the significant advantages of the algorithm proposed in this paper in improving the detection accuracy and lightweighting, providing an efficient and reliable detector for subsequent target tracking tasks.

**Fig 13:**
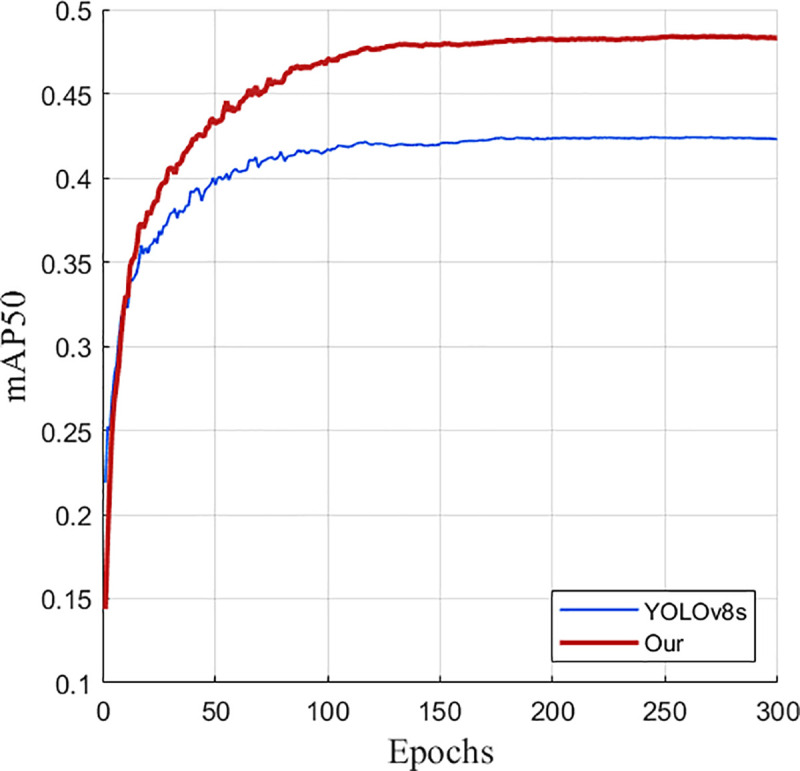
mAP50 comparison graph before and after improvement.

**Fig 14:**
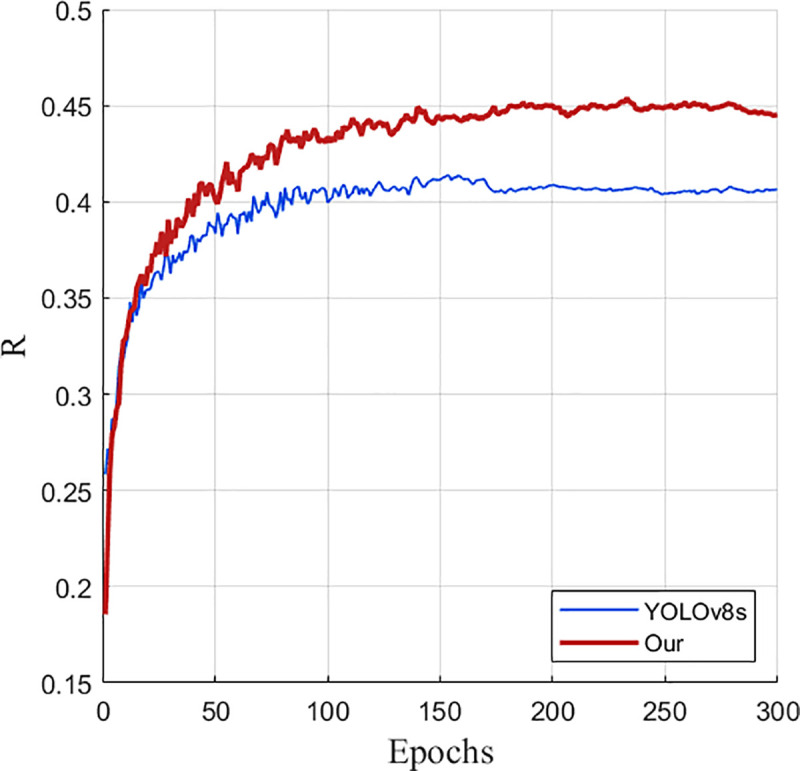
Recall rate comparison graph before and after improvement.

It can also be seen from the comparison graphs before and after the improvement in Fig 13 and Fig 14 that after 300 rounds of training of the training set pictures on the UAV data set by the improved model before and after the improvement, the average accuracy and recall rate of the improved model are much higher than those before and after the improvement, which intuitively demonstrates the advantages of the improved model.

**Fig 15:**
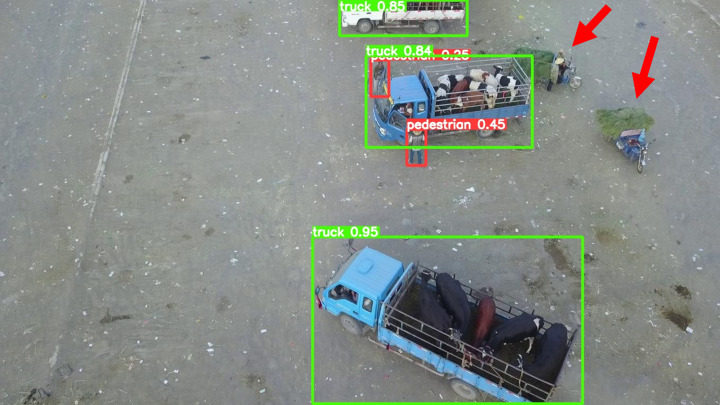
Detection results of the original YOLOv8s in open space.

**Fig 16:**
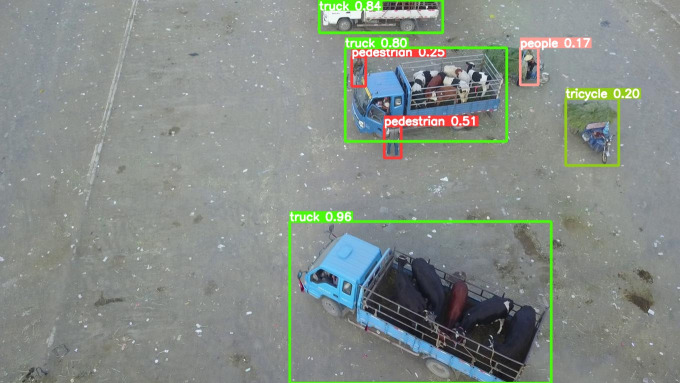
Detection results of the improved YOLOv8s in open space.

**Fig 17:**
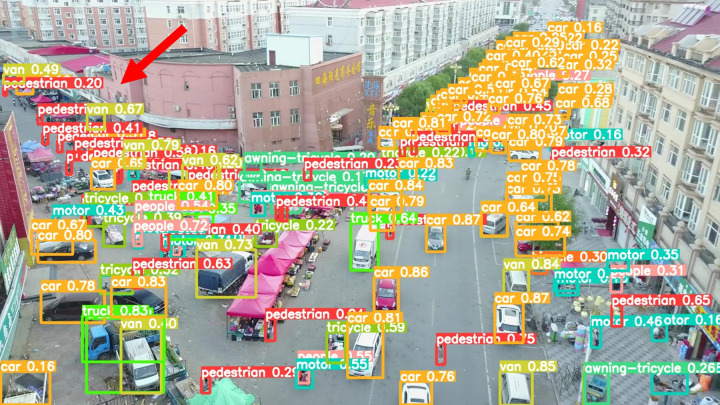
Detection results of the original YOLOv8s on densely parked roads.

**Fig 18:**
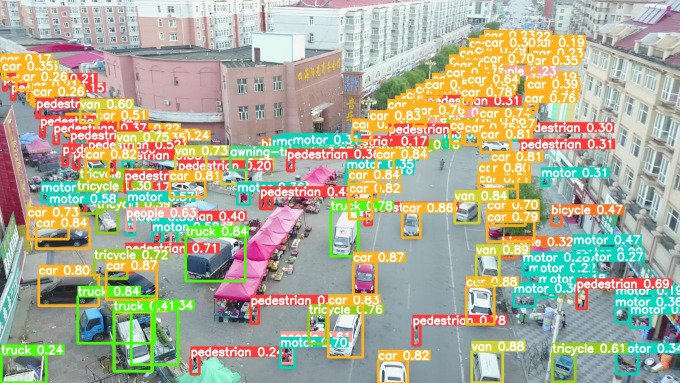
Detection results of the improved YOLOv8s on densely parked roads.

**Fig 19:**
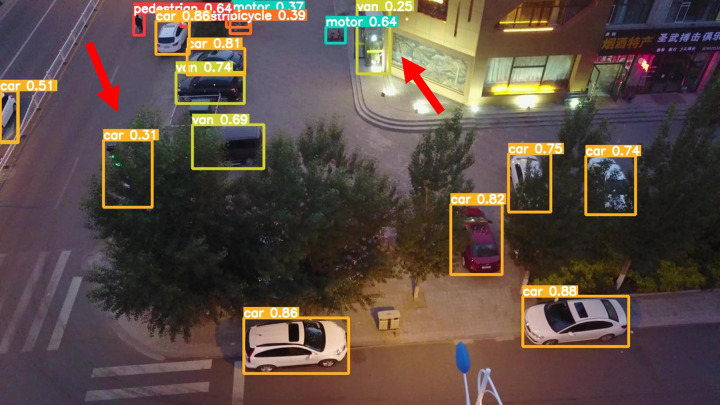
Detection results of the original YOLOv8s at low light intersections.

**Fig 20:**
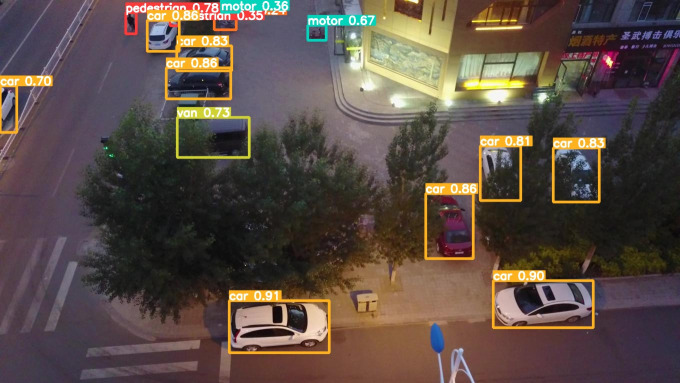
Detection results of the improved YOLOv8s at low light intersections.

The model before and after the improvement of YOLOv8s was tested on the verification set. The FIG shows the detection effects of the original YOLOv8s model and the improved YOLOv8s in different environments. Fig 15, Fig 17 and Fig 19 show the detection results of the original YOLOv8s. Fig 16, Fig 18 and Fig 20 show the detection results of the improved YOLOv8s.

It can be seen from Figs 15 and 16 that in the environment of a large open space, the red arrow in the FIG points to the missed targets of the original model, while the improved model can accurately detect the two targets of human and tricycle. In Fig 17 and Fig 18, there are multiple missed targets at the point of the red arrow in the FIG. The improved model can accurately identify many undetected vehicles in the upper left corner. In Fig 19 and Fig 20, two objects were mistakenly detected at the point pointed by the red arrow in the FIG. The improved model has higher detection accuracy and solves the problem of false detection. Therefore, the algorithm proposed in this paper can achieve efficient and accurate target detection in a variety of UAV shooting environments. Experimental results show that the algorithm can still maintain high detection accuracy and real-time performance under occlusion, illumination changes and other complex scenes, meeting the actual needs of UAV target detection in complex environments.

### 4.5 Comparative experiment

In order to better test the detection effect of the improved model proposed in this paper under the UAV data set, under the same experimental conditions, The improved YOLOv8s algorithm was compared with mainstream target detection algorithms such as YOLOv3-spp, YOLOv5s, YOLOv7tiny, YOLOv9c and YOLOv10s in the data set Visdrone2019. The experimental results are shown in Table 5. In Table 5, the performance indicators of each model in UAV detection tasks are listed, including detection accuracy, detection recall rate, average detection accuracy, parameter number, weight file size and detection speed.

**Table 5:** Comparative experiment.

Algorithm type	P(%)	R(%)	mAP50(%)	Params	Size(MB)	Speed(FPS)
YOLOv3-spp	50.6	39.8	39.9	62,594,983	125.7	63
YOLOv5s	43.5	33.5	30.3	7,078,183	14.4	83
YOLOv7tiny	48.0	39.0	36.8	6,031,950	12.3	139
YOLOv9c	54.8	39.9	42.5	13,070,044	27.0	42
YOLOv10s	51.9	39.7	40.4	8,042,700	16.6	256
Improved YOLOv8s	59.8	45.1	48.5	9,636,662	19.5	156

Table 5 shows the comparison of the six models in various performance. Compared with the other five mainstream detection algorithms, the improved YOLOv8s is the best in terms of detection accuracy, recall rate and average detection accuracy, and is superior to YOLOv3-spp and YOLOv9c in terms of parameter number and weight file size. And its detection speed is second only to that of YOLOv10s. In general, the improved YOLOv8s model has achieved significant improvements in both detection accuracy and recall rate, while also taking into account the lightweight requirements of the model.

**Fig 21:**
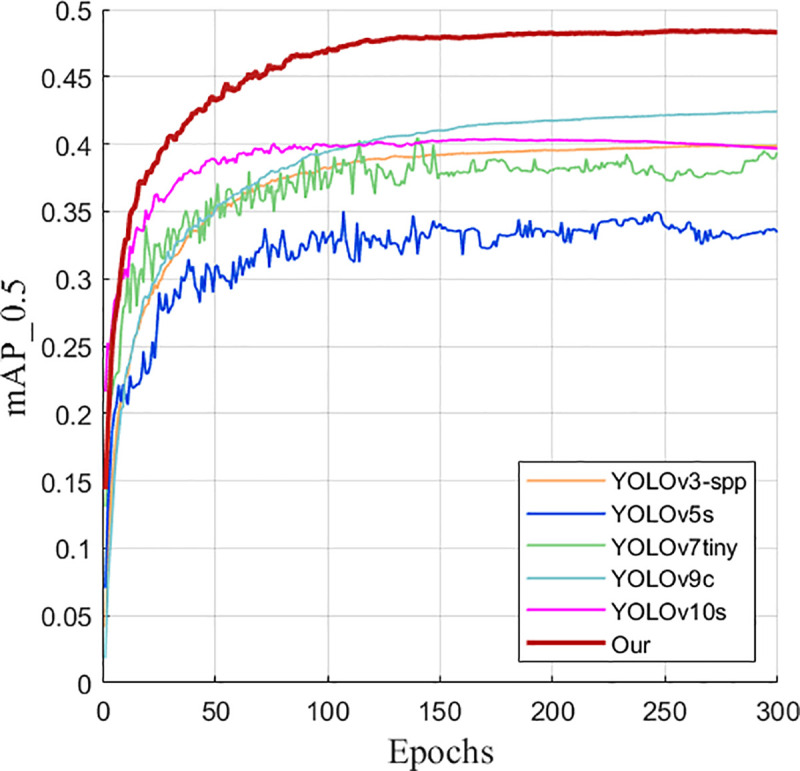
Comparison diagram of mAP50.

**Fig 22:**
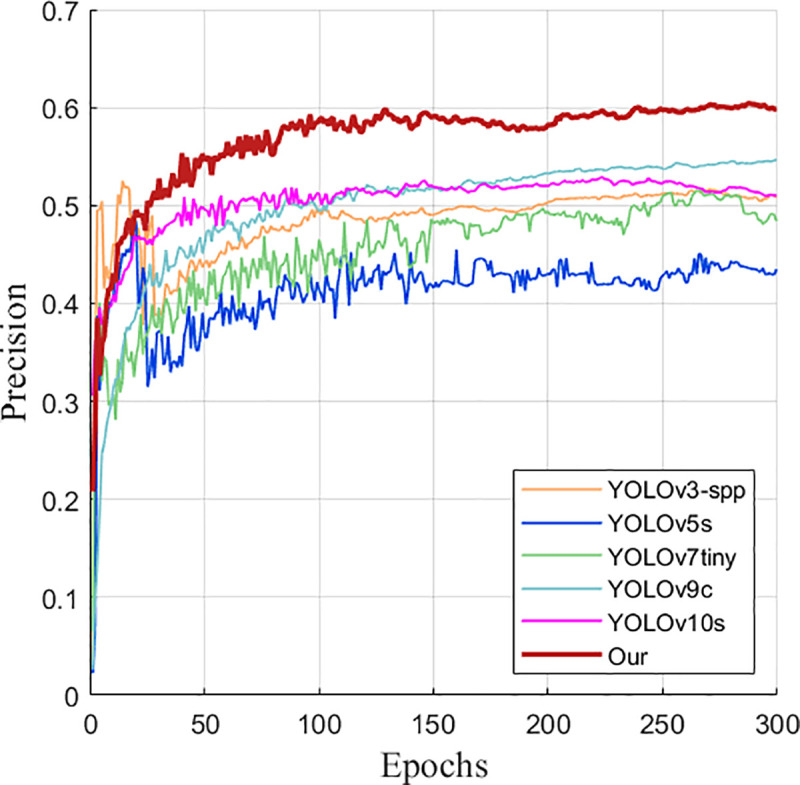
Comparison diagram of detection accuracy.

It can also be seen from the improved before and after comparison graphs in Fig 21 and Fig 22 that after 300 rounds of training on the training set pictures on the UAV data set, the average detection accuracy and detection accuracy of the improved YOLOv8s model are much higher than those of other six mainstream detection models.

**Fig 23:**
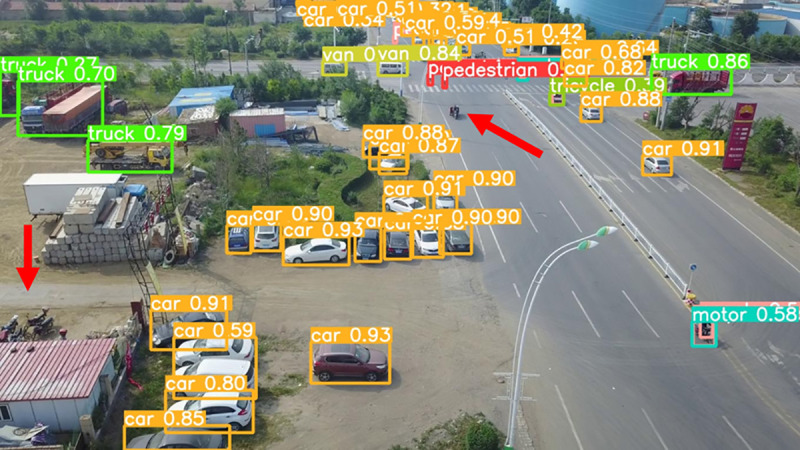
YOLOv3-spp.

**Fig 24:**
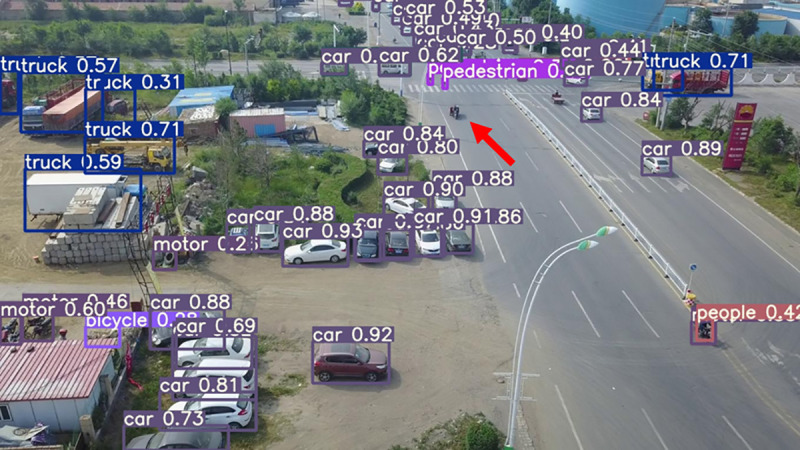
YOLOv5s.

**Fig 25:**
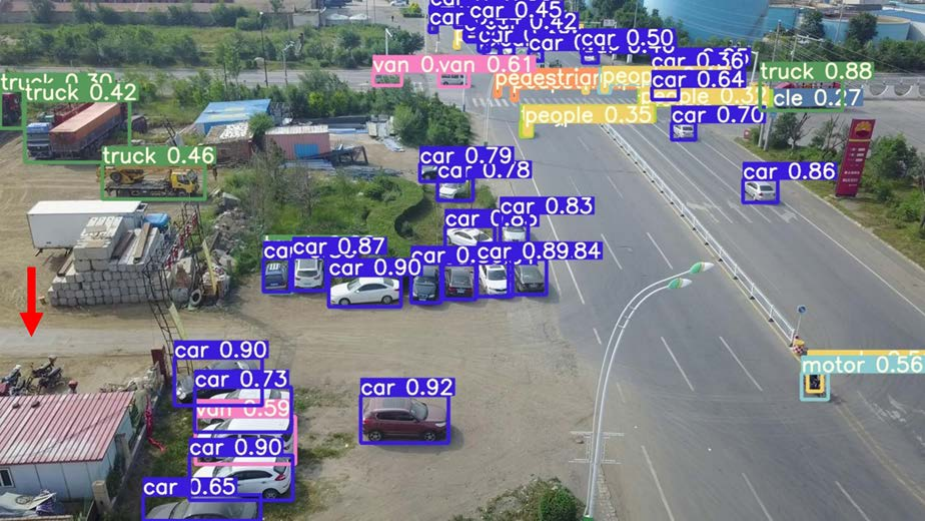
YOLOv7tiny.

**Fig 26:**
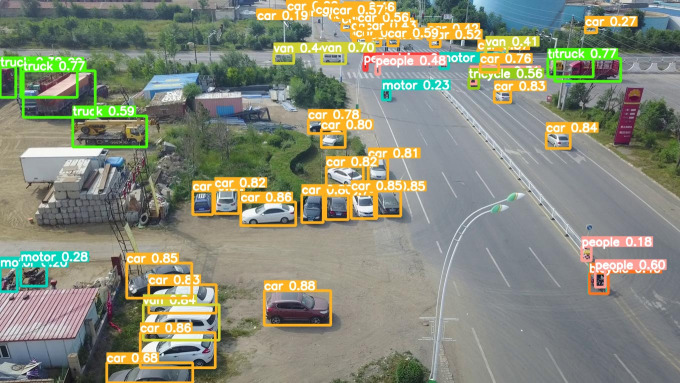
Improved YOLOv8s.

**Fig 27:**
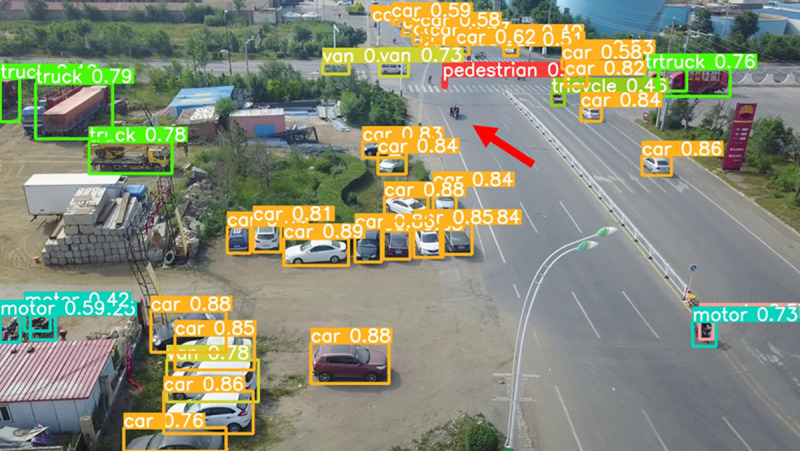
YOLOv9c.

**Fig 28:**
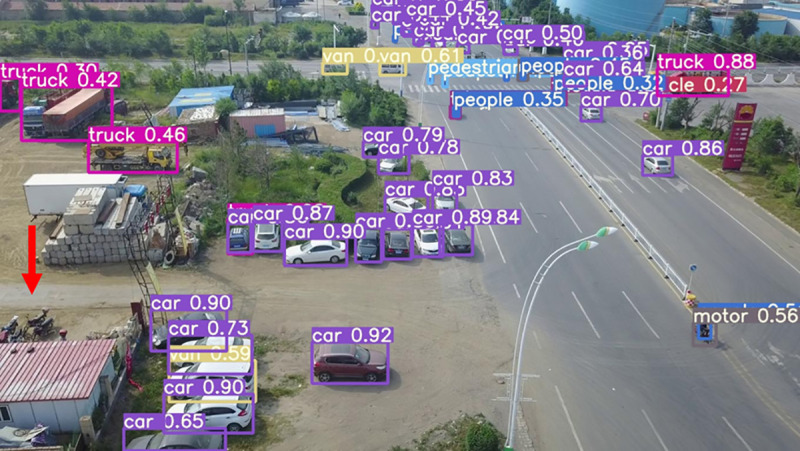
YOLOv10s.

Figs 23, 24, 25, 26, 27, and 28 respectively show the detection results of YOLOv3-spp, YOLOv5s, YOLOv7tiny, Improved YOLOv8s, YOLOv9c, and YOLOv10s.

As can be seen from Figs 23, 24, 25, 27, 28, YOLOv3-spp, YOLOv5s, YOLOv7tiny, YOLOv9c and YOLOv10s have different degrees of missing detection of the motorcycle target at the point indicated by the red arrow in the FIG. In Fig 26, the improved YOLOv8s proposed in this paper does not miss detection, indicating that the algorithm proposed in this paper is better in detection accuracy and more in line with the task of UAV target detection.

## 5. Sum up

The purpose of this paper is to solve the challenges of UAV in the field of target detection, especially the problems of target missing detection and lack of real-time performance in complex scenes. Uav is often faced with changing environment and dynamic targets when performing tasks, and traditional target detection algorithms are often difficult to cope with these complex situations. Therefore, in order to improve the speed and accuracy of target detection, This paper presents an improved object detection algorithm.

First of all, aiming at the limitations of YOLOv8s target detection algorithm in the aspects of insufficient detection accuracy of small targets, it is difficult to meet the needs of UAV for fast and accurate search in complex scenes, this paper proposes an improved target detection algorithm. In this algorithm, AKConv is introduced into C2F module, which makes the convolution operation more accurately adapt to the targets of different positions and scales. Then LSKA attention mechanism is introduced in SPPF module, which can not only capture the long range dependence effectively, but also improve the adaptability of features. In addition, the Bi-FPN feature pyramid network structure is introduced in the 18th layer of the model to accelerate and enrich the fusion of neck features, and combined with the SCDown structure, a new Bi-SCDown-FPN feature pyramid network structure is proposed, which can effectively improve the ability of target detection in complex environments. Experimental results show that the mAP of the improved algorithm on the VisDrone2019 UAV dataset is 48.5%, which is 5.1% higher than that of the original algorithm, and the number of parameters is 9.64M, which is 13.41% lower than that of the original algorithm. At the same time, comparative experiments are carried out on six models. In terms of detection accuracy, the improved YOLOv8s has certain advantages compared with the other five algorithms. Therefore, the improved algorithm proposed in this paper has a certain degree of improvement in speed and accuracy, and can meet the requirements of different tasks of UAVs in various rich scenarios.

This paper only studies the YOLOv8s detection algorithm model in the YOLOv8 series. In the future, we can consider the study of more complex models such as YOLOv8l, so as to improve the performance of the target detection algorithm in more complex scenes, especially in the case of higher resolution or larger target detection, so as to further improve the detection accuracy and robustness. The data set VisDrone2019 used in this paper is a recognized UAV data set. In the future, we can consider using other recognized UAV data sets or homemade UAV data sets to enhance the richness and authenticity of this study, further verify the generalization ability of the algorithm, and improve the adaptability of the algorithm in real applications.

## Supporting information

S1 Data (ZIP)
